# Assessing predictors of delayed antenatal care visits in Rwanda: a secondary analysis of Rwanda demographic and health survey 2010

**DOI:** 10.1186/1471-2393-14-290

**Published:** 2014-08-28

**Authors:** Anatole Manzi, Fabien Munyaneza, Francisca Mujawase, Leonidas Banamwana, Felix Sayinzoga, Dana R Thomson, Joseph Ntaganira, Bethany L Hedt-Gauthier

**Affiliations:** Partners In Health, Kigali, Rwanda; College of Medicine and Health Sciences, University of Rwanda, Kigali, Rwanda; Development Alternatives Incorporated, United States Agency for International Development, Kigali, Rwanda; Ministry of Health, Government of Rwanda, Kigali, Rwanda; Department of Global Health and Social Medicine, Harvard Medical School, Boston, USA; Partners In Health, Boston, USA

**Keywords:** Antenatal care, Delayed, Demographic survey, Rwanda, Predictors

## Abstract

**Background:**

Early initiation of antenatal care (ANC) can reduce common maternal complications and maternal and perinatal mortality. Though Rwanda demonstrated a remarkable decline in maternal mortality and 98% of Rwandan women receive antenatal care from a skilled provider, only 38% of women have an ANC visit in their first three months of pregnancy. This study assessed factors associated with delayed ANC in Rwanda.

**Methods:**

This is a cross-sectional study using data collected during the 2010 Rwanda DHS from 6,325 women age 15–49 that had at least one birth in the last five years. Factors associated with delayed ANC were identified using a multivariable logistic regression model using manual backward stepwise regression. Analysis was conducted in Stata v12 applying survey commands to account for the complex sample design.

**Results:**

Several factors were significantly associated with delayed ANC including having many children (4–6 children, OR = 1.42, 95% CI: 1.22, 1.65; or more than six children, OR = 1.57, 95% CI: 1.24, 1.99); feeling that distance to health facility is a problem (OR = 1.20, 95% CI: 1.04, 1.38); and unwanted pregnancy (OR = 1.41, 95% CI: 1.26, 1.58). The following were protective against delayed ANC: having an ANC at a private hospital or clinic (OR = 0.29, 95% CI: 0.15, 0.56); being married (OR = 0.85, 95% CI: 0.75, 0.96), and having public mutuelle health insurance (OR = 0.81, 95% CI: 0.71, 0.92) or another type of insurance (OR = 0.33, 95% CI: 0.23, 0.46).

**Conclusion:**

This analysis revealed potential barriers to ANC service utilization. Distance to health facility remains a major constraint which suggests a great need of infrastructure and decentralization of maternal ANC to health posts and dispensaries. Interventions such as universal health insurance coverage, family planning, and community maternal health system are underway and could be part of effective strategies to address delays in ANC.

## Background

In most developing countries, limited progress towards Millennium Development Goal 5 (75% reduction in the maternal mortality ratio between 1990 and 2015) reflects poor quality health services and socioeconomic challenges which limit access to health care [1]. According to the WHO, 358,000 women died in 2008 and the majority (87%) of deaths occurred in sub-Saharan Africa [2]. Leading causes of maternal deaths, including hemorrhaging, anemia, and hypertension during pregnancy [3], could be averted if detected early.

Utilization of maternal health services is associated with improved pregnancy outcomes [4], including reduced maternal and perinatal mortality [5–8]. When mothers receive prenatal care during the first trimester, early signs of pregnancy complications such as anemia, hypertension, hyperemesis gravidarum, polyhydramnios, ante-partum hemorrhage, gestational diabetes, and urinary tract infections can be detected [9, 10]. However, utilization of antenatal care (ANC) services are often limited or delayed in developing countries due to demographic, education, culture, and economic factors and geographic barriers [11–13].

Rwanda has achieved a remarkable decline in maternal mortality from 750 to 476 deaths per 100,000 within only five years (2005–2010) [14]. This success is credited to improved health system strengthening through cross-sector collaborations, community-based care, evidence-based policy making, strong partnership with local and central governments and a high level political commitment [15]. As a result, 98% of pregnant women received at least one ANC from a skilled provider in 2010 [14]. Although this is a great success, Rwanda’s maternal mortality rate remains in the highest 20% worldwide [16] and only 38% of women have their first ANC visit in the first three months of pregnancy [14]. In the context of progressive health systems strengthening and economic improvement, barriers still remain for timely uptake of ANC care. The goal of this research is to understand factors associated with delayed ANC in Rwanda to better understand limited improvement in this domain.

## Methods

The 2010 RDHS is a nationally representative two-stage cluster sample that included 492 primary sampling units (PSUs) and 12,540 households. Data collection occurred between September 26, 2010 and March 10, 2011. Respondents answered detailed questions about their reproductive health histories, reproductive health practices, recent pregnancy experiences, household assets, and access to health services [17]. This study only includes the 6,325 women ages 15–49 years who had a pregnancy in the last five years [14]. If there was more than one pregnancy in the last five years, the outcomes and predictors were based on their last pregnancy.

**Figure 1:**
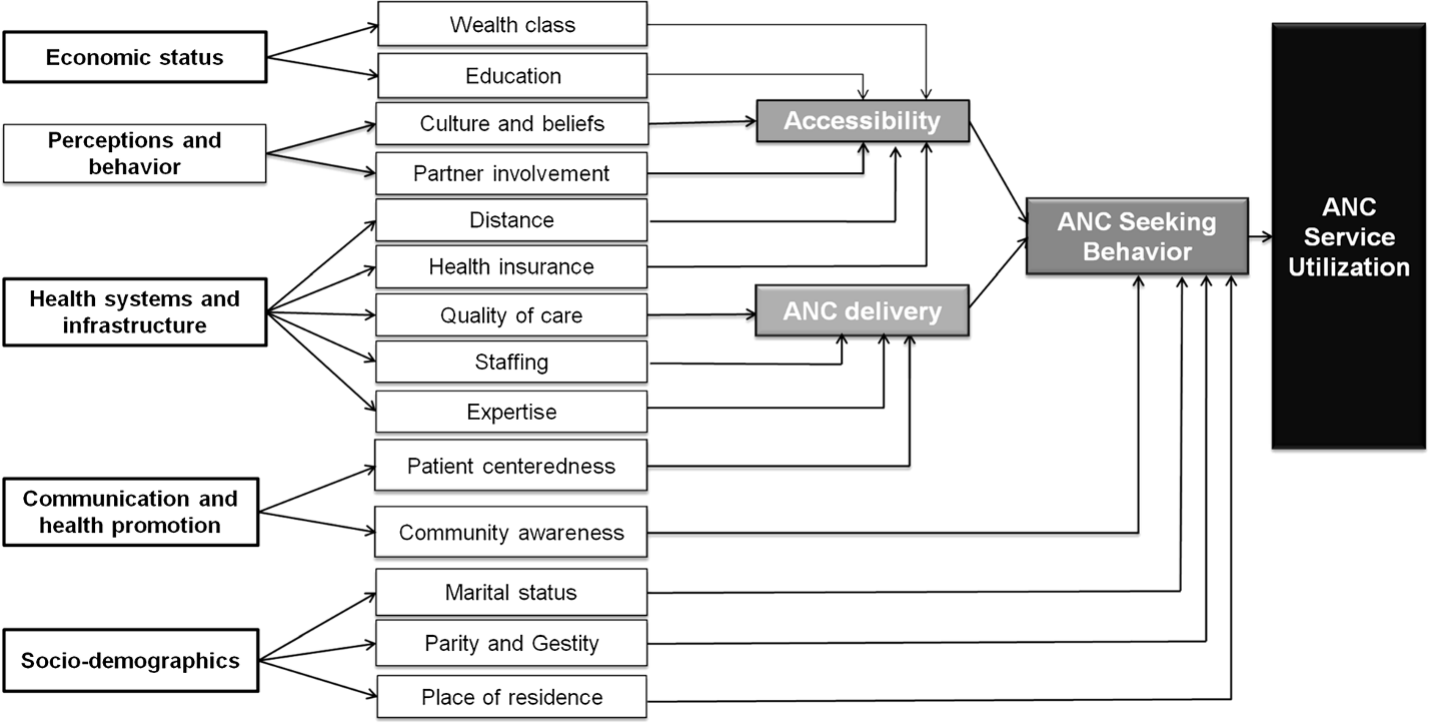
**Conceptual framework.**

The primary outcome for this study is delayed ANC, defined as having no ANC visit or having the first ANC visit during the second or third trimester of pregnancy. Based on a conceptual framework (Figure 1), 16 potential predictors of delayed ANC collected in the 2010 DHS were identified: number of children, place of residence, place of ANC, marital status, having health insurance, problem with distance to clinic, unwanted pregnancy, woman’s age, wealth status, woman’s education, partner’s education, woman’s employment status, partner’s employment status, knowledge of ovulatory cycle, and access to TV or radio at least once a week. Due to collinearity, partner’s education and working status were combined into a single variable.

Variables that were differentially distributed among women who did and did not have delayed ANC (p < 0.05 in the design-adjusted Chi-squared) were retained for model building. Collinearity was assessed, and for covariates that were identified to be strongly collinear (r > =0.8, using Pearson’s correlation test) the variable more strongly correlated with delayed ANC was retained. Manual backward stepwise regression was used to develop a multivariable logistic regression model of predictors of delayed ANC in Rwanda. Only factors significant at the α = 0.05 level were retained in the final model except age and place of residence which were considered by the study team as potential confounders. Analysis was completed in Stata v12, with svyset commands to apply inverse probability weights that account for oversampling of urban PSUs, and to adjust for clustering of observations within PSUs and stratification by district. Odds ratios (ORs) and 95% confidence intervals are reported.

## Ethical statement

This study is a secondary analysis of the 2010 Rwanda Demographic Health Survey and as such, no ethical approval was required. We registered and requested for access to data from DHS on-line archive and received an approval to access and download de-identified DHS data files. All guidelines, including treating data as confidential and not making effort to identify individual respondents, were respected.

## Results

Of the 6,325 women that had a pregnancy in the last 5 years, 6,211 attended a health facility for their first ANC during the last pregnancy. Among the 6,211 women who presented to ANC clinics, 3,797 women (61.1%, 95% CI: 59.3%, 62.7%) had a delayed first visit (Table 1). In bivariate analysis, the following factors were significantly associated with delayed ANC: number of children (p < 0.001), area of residence (p = 0.018), place of ANC (p < 0.001), marital status (p = 0.012), type of health insurance (p < 0.001), expressed problem with distance to health facility (p = 0.002), unwanted pregnancy (p < 0.001), age (p < 0.001), wealth status (p < 0.001), woman’s education level (p < 0.001), partner’s education level (p < 0.001), woman’s employment status (p < 0.001), partner’s employment status (p < 0.001), and access to TV or radio at least once a week (p = 0.003). Also, combining marital, education and employment status, we found that having an employed partner with at least secondary education was associated with delayed ANC.

**Table 1:** **Bivariate relationships between demographic characteristics and delayed antenatal care in Rwanda**

Characteristic	N	% [95% CI]		P-value
Age group				<0.001
15-24	1,251	56.8	[53.6,60.1]	
25-34	3,235	60.3	[58.2,62.4]	
35-44	1,526	65.2	[62.5,67.8]	
45+	269	65.6	[59.7,71.2]	
**Residence**				0.018
Urban	806	55.9	[51.4,60.4]	
Rural	5475	61.8	[60.0,63.6]	
**Number of children**				<0.001
1-3	3,571	56.6	[54.5,58.6]	
4-6	1,850	66.0	[63.4,68.5]	
7+	860	68.9	[65.4,72.2]	
**Place of antenatal care**				<0.001
Health center	5,799	61.6	[59.9,63.3]	
Home or other	20	67.0	[49.3,80.9]	
Dispensary/health post	103	69.9	[58.6,79.2]	
District hospital	239	56.3	[48.2,64.1]	
Private hospitals/clinics	56	19.8	[10.7,33.5]	
Referral hospitals	53	40.4	[28.6,53.3]	
**Married**				0.012
No	2682	63.0	[60.7,65.3]	
Yes	3599	59.5	[57.5,61.5]	
**Type of health insurance**				<0.001
No	1,657	66.6	[63.9,69.2]	
Mutuelle	4402	60.2	[58.4,62.0]	
RAMA_MMI and others	194	31.2	[24.3,39.1]	
**Problem with distance**				0.002
No	4577	59.6	[57.8,61.5]	
Yes	1702	64.8	[61.9,67.6]	
**Pregnancy**				<0.001
Wanted	3600	56.8	[54.7,58.9]	
Unwanted	2679	66.8	[64.7,68.8]	
**Wealth index**				<0.001
Poorest	1427	64.2	[61.2,67.1]	
Poorer	1335	62.7	[59.9,65.5]	
Middle	1230	62.1	[58.8,65.4]	
Richer	1177	62.0	[58.9,64.9]	
Richest	1112	52.7	[49.3,56.1]	
**Women education secondary or higher**				<0.001
No	5665	62.3	[60.6,64.0]	
Yes	616	49.4	[45.3,53.5]	
**Husband with secondary or higher education**				<0.001
No	5,150	62.4	[60.6,64.1]	
Yes	672	49.5	[45.3,53.7]	
Not married	457	62.9	[57.8,67.9]	
**Women’s employment status**				<0.001
Informal sector/not working	5,668	62.3	[60.5,64.0]	
Formal sector	605	50.0	[45.4,54.6]	
**Partner’s employment status**				<0.001
Informal sector/not working	4,496	62.4	[60.5,64.3]	
Formal sector	1,316	55.5	[52.4,58.6]	
Not married	457	62.9	[57.8,67.9]	
**Current marital status**				0.277
Never in union	457	62.9	[57.8,67.9]	
Married/living with partner	5,234	60.6	[58.8,62.4]	
Widowed and/or separated	590	63.7	[59.7,67.5]	
**Husband’s education and employment status**				
Not married	457	62.9	[57.8,67.9]	0.003
Employed partner with secondary education	413	45.3	[40.0,50.6]	
Employed partner without secondary education	903	60.2	[56.8,63.5]	
Unemployed partner with secondary education	257	56.1	[49.5,62.5]	
Unemployed partner without secondary education	4,239	62.8	[60.9,64.7]	
**Knowledge of menstrual cycle**				0.072
Yes	768	57.8	[53.8,61.7]	
No	5,510	61.5	[59.7,63.2]	
**TV or radio at least once a week**				0.003
No	2,114	63.8	[61.4,66.1]	
Yes	4,161	59.7	[57.7,61.6]	

Table 2 contains results of the full and reduced (only including factors identified as significant using the backwards stepwise regression) models. In the reduced model, several factors were associated with delayed ANC: having 4–6 children (OR = 1.42, 95% CI: 1.22, 1.65) or more than 6 children (OR = 1.57, 95% CI: 1.24, 1.99) versus 1–3 children; feeling that distance to health facility is a problem (OR = 1.20, 95% CI: 1.04, 1.38); and having an unwanted pregnancy (OR = 1.41, 95% CI: 1.26, 1.58). Different factors were associated with receiving ANC during the first trimester: having an ANC at a private hospital or clinic (OR = 0.29, 95% CI: 0.15,0.56) versus a public health center; being married (OR = 0.85, 95% CI: 0.75, 0.96), and having public mutuelle health insurance (OR = 0.81, 95% CI: 0.71, 0.92) or another type of insurance (OR = 0.33, 95% CI: 0.23, 0.46) versus no insurance.

**Table 2:** **Multivariate logistic regression model with odds rations, P-value and confidence intervals for Women’s delay to the first antenatal care visits in Rwanda**

	Full				Reduced			
Characteristics	OR	*P*	95% CI		OR	*P*	95% CI	
**Age group**								
15-24	1.00				1.00			
25-34	1.14	0.107	0.97	1.34	1.13	0.120	0.97	1.33
35-44	1.08	0.462	0.87	1.34	1.08	0.486	0.87	1.33
45+	0.94	0.741	0.67	1.33	0.94	0.732	0.67	1.33
**Place of residence**								
Urban	1.00				1.00			
Rural	0.87	0.245	0.68	1.10	1.03	0.760	0.84	1.26
**Number of children**								
1-3	1.00				1.00			
4-6	1.41	<0.001	1.22	1.65	1.42	<0.001	1.22	1.65
7+	1.55	<0.001	1.22	1.97	1.57	<0.001	1.24	1.99
**Place of antenatal care**								
Health center	1.00				1.00			
Home or other	2.10	0.137	0.79	5.57	1.63	0.200	0.77	3.47
Dispensary/health post	1.50	0.118	0.90	2.48	1.47	0.130	0.89	2.43
District hospital	0.96	0.808	0.69	1.33	0.94	0.734	0.68	1.32
Private hospitals/clinics	0.33	0.001	0.17	0.65	0.29	<0.001	0.15	0.56
Referral hospitals	0.72	0.200	0.43	1.19	0.65	0.093	0.40	1.07
**Married**								
No	1.00				1.00			
Yes	0.86	0.028	0.76	0.98	0.85	0.012	0.75	0.96
**Type of health insurance**								
No	1.00				1.00			
Mutuelle^†^	0.81	0.003	0.71	0.93	0.81	0.002	0.71	0.92
RAMA, MMI, and others^‡^	0.43	<0.001	0.29	0.64	0.33	<0.001	0.23	0.46
**Problem with distance to health facility**								
No	1.00				1.00			
Yes	1.19	0.017	1.03	1.37	1.20	0.012	1.04	1.38
**Pregnancy**								
Wanted	1.00				1.00			
Unwanted	1.41	<0.001	1.25	1.58	1.41	<0.001	1.26	1.58
**Wealth index**								
Poorest	1.00							
Poorer	0.97	0.707	0.82	1.14				
Middle	0.99	0.951	0.82	1.20				
Richer	0.99	0.900	0.81	1.21				
Richest	0.86	0.254	0.67	1.11				
**Women’s education secondary or higher**								
No	1.00							
Yes	0.94	0.565	0.75	1.17				
**Women’s employment status**								
Informal sector/not working	1.00							
Formal sector	0.84	0.100	0.68	1.03				
**Partners' education employment status**								
Not married	1.00							
Employed partner with secondary education	0.76	0.156	0.53	1.11				
Employed partner without secondary education	0.98	0.889	0.74	1.30				
Unemployed partner with secondary education	0.91	0.593	0.63	1.30				
Unemployed partner without secondary education	0.97	0.814	0.76	1.25				
**Knowledge of menstrual cycle**								
Yes	1.00							
No	1.00	0.957	0.84	1.20				
**TV or radio at least once a week**								
No	1.00							
Yes	0.98	0.720	0.86	1.11				

^*†*^ *Community based health insurance scheme,* ^*‡*^ *Alternative or private health insurance schemes.*

## Discussion

In this secondary analysis of the 2010 RDHS, we found that more than three-fifths of pregnant women presented late for their first ANC visit. Having many children, poor geographic access to health facility and unwanted pregnancy were associated with the probability of a woman to not have an ANC visit in the first trimester. Significant delays for the first ANC visit have been observed in other countries in the region, including Ethiopia (more than half of women had a delayed ANC in 2012) [13]. In other studies, age at delivery, family income, media exposure, attitude towards pregnancy, knowledge about the danger signs of pregnancy, husband’s approval of ANC, and distance to health facility were associated with ANC service utilization at any point during pregnancy [13, 18]. In 2011, Hagey and colleagues explored social and behavioral factors that affect timely initiation of ANC from the perspective of health care providers in Kigali city. They found that women’s knowledge gaps; having previous births; limited involvement of male partners; problems with health insurance; and ANC culture were the main barriers to first ANC initiation [19].

For this study, some variables such as quality of antenatal care provided, and women’s perceptions of the ANC were not available for consideration in the existing RDHS data, but are likely important factors in determining timing of ANC. Of the available data, some factors did not appear significant in our analyses, most notably income. Other studies have found that women’s poverty limits access to maternal health services, including ANC [12, 20–24]; however, in Rwanda, the availability and uptake of health insurance may offset the impact of income on delays in care seeking behavior. In our study, almost all women (91.2%) had health insurance, and women with no health insurance were more likely to delay their first antenatal care visit than women with insurance.

In our analysis, having more than four children was significantly associated with delayed ANC [14]. Promotion of accessible family planning methods at the community level could help couples to achieve their desired number of children, educate them about the benefits of birth spacing, and promote better health seeking behavior during pregnancy, all of which are associated with improved child survival and reduced total number of pregnancies [25]. The majority of women in this study (61%) did not know about their menstrual cycle which suggests a lack of information about reproductive health broadly including information about the benefits of timely ANC, a finding that is consist with other studies [26]. Raising awareness about reproductive health is demonstrated to improve maternal health service utilization [3, 27].

Surprisingly, our multivariable analysis did not include age as a risk factor for delayed ANC. This differs from recent studies that reported a significant association between delayed or lack of maternal health service utilization and older age of women [13, 28, 29]. More detailed studies are needed to better understand the factors that mitigate the effect of age on uptake of services in Rwanda.

We anticipated that delayed ANC would be lower among women seeking care from health posts because in Rwanda, health posts are more geographically accessible by pregnant women than other types of health facilities. One potential reason that health posts are not associated with timely ANC is that they are not yet accredited and equipped to deliver formal ANC. In this context, health post nurses do not vest much effort in monitoring of pregnant women. We also found that women who noted distance as a barrier to care were significantly more likely to have delayed ANC. Formally decentralizing ANC services to health posts could improve early uptake of ANC; this proposal is supported by results from other studies [30]. Decentralization of ANC to community health posts and capacitating health posts and dispensaries to provide prenatal and postnatal services would decrease the distances that women must travel to get ANC services. However, measures such as clinical mentorship programs or supervision would be needed to ensure high quality of care at decentralized sites.

Rwanda is one of the sub-Saharan African countries that promote male involvement in antenatal care, preventing mother-to-child transmission of HIV and other maternal and child health services. With this policy, pregnant women are more likely than other settings to be accompanied by a partner for ANC. This might have caused some delays in ANC service utilization. Further studies are needed to understand the effects of this policy to the timing of ANC on married women, as well as among unmarried, separated or pregnant women living far from their male partners.

There are several limitations to this study. First, since this was a secondary analysis of the 2010 RDHS, we did not have all the variables proposed in our conceptual framework. We could not explore associations between the quality of care at the nearest health facility on delayed ANC. In addition, recall bias, particularly about older pregnancies, may have affected our secondary analysis. Moreover, the analysis included the primary predictors of the study outcome. Therefore, future researchers in this domain should explore whether or not the effect of these risk factors varies by different population strata.

Recent studies have shown that poor quality of care, and insufficient medical equipment and infrastructure for maternal health services contribute to limited maternal and child health services utilization [31–33]. This study measured predictors of delaying the first antenatal care visit. Therefore, further studies should assess risk factors for delays in subsequent visits.

Furthermore, the RDHS has limited information on perception and acceptability of ANC services, which is an important area for further study. Finally, the RDHS did not include questions on travel time to the clinic, and the only variable that could be included was a question that measured the perceived burden of distance on access.

## Conclusion

The study assessed predictors of delayed ANC (receipt of first ANC in the second or third trimester of pregnancy) in Rwanda. We found that several socio-demographic factors was associated with delayed ANC, so we propose specific interventions to reduce these barriers. Distance to health facility remains a major barrier to ANC services which suggests a potential for decentralization of maternal ANC to health posts and dispensaries. There is a need to study the effectiveness of the existing community outreach programs such as community-based family planning and immunization as well as the feasibility of ANC decentralization and integration within other community-based interventions.

Several countries including Rwanda have launched an automatic phone text message system (RapidSMS) to remind community health workers about women’s appointment [34]. Rwanda has also initiated semiannual maternal, child and adolescent health weeks with activities coordinated in communities by community health workers. We believe that these initiatives can promote better health seeking behavior and, combined with decentralized ANC, could increase access and minimize delays in ANC service utilization. However, further studies are required to measure effects of these synergetic interventions.

Current efforts to provide universal coverage of health insurance, access to family planning, and strengthened community maternal health systems are important strategies for linking women to ANC care early in their pregnancies. In addition to the existing programs, though, these findings suggest a need for more infrastructure enabling geographic access to services. Furthermore, health promotion campaigns in communities with an emphasis on maternal health would additionally ensure that most women gain access to basic reproductive health information. Formal or ad hoc trainings and workshops should be implemented in communities to explain basic knowledge about reproductive health and the importance of antenatal service utilization.

## Abbreviations

ANC: Antenatal care
OR: Odd ratio
CI: Confidence interval
RDHS: Rwanda demographic and health survey
WHO: World Health Organization.
